# Epigenetic changes in patients with post-acute COVID-19 symptoms (PACS) and long-COVID: A systematic review

**DOI:** 10.1017/erm.2024.32

**Published:** 2024-10-22

**Authors:** Madhura Shekhar Patil, Emma Richter, Lara Fanning, Jolien Hendrix, Arne Wyns, Laura Barrero Santiago, Jo Nijs, Lode Godderis, Andrea Polli

**Affiliations:** 1 Centre for Environment and Health, Department of Public Health and Primary Care, KU Leuven, Leuven, Belgium; 2 Pain in Motion (PAIN) Research Group, Department of Physiotherapy, Human Physiology, and Anatomy, Vrije Universiteit Brussel, Brussels, Belgium; 3 Research Foundation, Flanders (FWO); 4 Department of Cell Biology, Genetics, Pharmacology and Histology – University of Valladolid, Spain; 5 Department of Health and Rehabilitation, Institute of Neuroscience and Physiology, University of Gothenburg, Gothenburg, Sweden; 6 External Service for Prevention and Protection at Work (IDEWE), Leuven, Belgium

**Keywords:** DNA methylation, epigenetics, long-COVID, miRNAs, PACS

## Abstract

**Background:**

Up to 30% of people infected with SARS-CoV-2 report disabling symptoms 2 years after the infection. Over 100 persistent symptoms have been associated with Post-Acute COVID-19 Symptoms (PACS) and/or long-COVID, showing a significant clinical heterogeneity. To develop effective, patient-targeted treatment, a better understanding of underlying mechanisms is needed. Epigenetics has helped elucidating the pathophysiology of several health conditions and it might help unravelling inter-individual differences in patients with PACS and long-COVID. As accumulating research is exploring epigenetic mechanisms in PACS and long-COVID, we systematically summarized the available literature on the topic.

**Methods:**

We interrogated five databases (Medline, Embase, Web of Science, Scopus and medXriv/bioXriv) and followed PRISMA and SWiM guidelines to report our results.

**Results:**

Eight studies were included in our review. Six studies explored DNA methylation in PACS and/or long-COVID, while two studies explored miRNA expression in long-COVID associated with lung complications. Sample sizes were mostly small and study quality was low or fair. The main limitation of the included studies was a poor characterization of the patient population that made a homogeneous synthesis of the literature challenging. However, studies on DNA methylation showed that mechanisms related to the immune and the autonomic nervous system, and cell metabolism might be implicated in the pathophysiology of PACS and long-COVID.

**Conclusion:**

Epigenetic changes might help elucidating PACS and long-COVID underlying mechanisms, aid subgrouping, and point towards tailored treatments. Preliminary evidence is promising but scarce. Biological and epigenetic research on long-COVID will benefit millions of people suffering from long-COVID and has the potential to be transferable and benefit other conditions as well, such as Myalgic Encephalomyelitis/Chronic Fatigue Syndrome (ME/CFS). We urge future research to employ longitudinal designs and provide a better characterization of included patients.

## Introduction

COVID-19 is caused by Severe Acute Respiratory Syndrome Coronavirus 2 (SARS-CoV-2) (Ref. 1). Most individuals infected with SARS-CoV-2 suffer from a mild disease but some patients develop moderate-to-severe disease and require hospitalization (Refs 2, 3). Though the majority of cases recover within the first 2-3 weeks, accumulating clinical observations show that a significant proportion of patients with COVID-19 develop persistent symptoms (Refs 2, 3, 4). Up to 30% of people infected with SARS-CoV-2 during the first wave reported disabling symptoms, affecting everyday activities, 2 years after the infection (Ref. 4). Over 100 persistent symptoms have been associated with COVID-19, which led to coining the term post-acute COVID-19 symptoms (PACS) and long-COVID. PACS refers to symptoms persisting over 4 weeks after infection, while long-COVID is used when symptoms persist for 12 weeks or longer, and cannot be explained by alternative diagnosis (Ref. 5). The most common symptoms are fatigue, post-exertional symptom exacerbation (PESE), pain, and cognitive problems – e.g. concentration difficulties and brain fog (Refs 6, 7). Long-COVID represents a huge burden for these patients and a significant cost for healthcare systems worldwide (Refs 8, 9, 10).

One crucial advancement to the understanding of long-COVID is the observation that different subgroups likely exist. Subgroups are probably not linked to the characteristics of the virus or its variants, but rather to individual characteristics. While some factors such as obesity, depression and the presence of comorbidities are risk factors for both acute COVID-19 and long-COVID (Refs 11, 12, 13, 14) others are specific to either the acute or chronic phase. Severity and mortality in acute COVID-19 was associated with older age and male sex (Ref. 11). On the contrary, risk factors for long-COVID include female sex and younger age, suggesting that gender differences can be very relevant (Refs 12, 13, 14). Importantly, long-COVID can develop after very mild symptoms at onset (Ref. 15). A study on 545 patients found that over 70% of people with persistent symptoms started with mild or moderate initial infection (Ref. 16). This suggests that mechanisms of long-COVID might be different from the ones in the acute phase. Findings from research on COVID-19 should not be automatically generalized to long-COVID, and specific research is needed.

The acute phase of COVID-19 has been extensively studied, in the quest for mechanisms that can then be targeted by specific treatments. Hyperinflammation and coagulopathy as a result of the dysregulated immune response play a role in the severity of the disease (Ref. 17). Epigenetics is the study of phenotype changes without changes in the DNA sequence and includes alterations in DNA methylation, histone modifications, chromatin reorganization, miRNAs and lncRNAs (Ref. 18). It has been shown to be involved in the pathophysiology of many diseases and led to breakthrough findings in understanding a treatment of neurological disorders and cancer (Ref. 19). Epigenetic modifications have been linked to the aberrant immune response and have been suggested to predict disease severity, symptoms persistence and poor prognosis (Ref. 20). For instance, Genome-wide DNA methylation in peripheral blood mononuclear cells from severe COVID-19 patients showed hypermethylation of interferon-related genes and hypomethylation of cytokine genes, suggesting a potential role for sustained inflammation (Ref. 21). Other epigenome-wide DNA methylation association studies of peripheral blood samples of COVID-19 patients revealed an association between the increased severity of COVID-19 and altered DNA methylation at genes involved in inflammasome-related pathways, major histocompatibility factor, and HLA-C involved in the interferon-response pathway (Ref. 22). Similarly, non-coding RNA interference exerted by long non-coding RNAs (lncRNAs) or micro RNAs (miRNAs) (another set of mechanisms responsible for post-transcriptional regulation of gene expression) also show changes during COVID-19 infection (Refs 23, 24). Hundreds of lncRNAs and miRNAs show significant differential expression which in turn predict severity of the infection and/or mortality (Refs 25, 26, 27). Regulation of miRNAs and lncRNAs seem to help to relieve acute symptoms via downregulation of pro-inflammatory cytokines (Refs 28, 29). Taken together, the aforementioned observations show clear changes in epigenetic signatures of patients with acute COVID-19.

These findings suggest that epigenetic biomarkers might help unravelling inter-individual differences, disease presentation and prognosis in patients with PACS and long-COVID. Research on epigenetic changes and non-coding RNA interference is scarce in long-COVID, but preliminary observations are accumulating. These observations suggest that long-COVID can be explained by an intricate set of epigenetic mechanisms. For all these reasons, a systematic summary of the current evidence is warranted, but is currently unavailable and represents an important knowledge gap. Here we report a systematic review aiming to summarize the available literature exploring epigenetic changes associated with PACS or long-COVID.

## Materials and methods

The protocol for this systematic review was registered on PROSPERO (registration ID: CRD42023393690) and the review was conducted in accordance with the 2020 Preferred Reporting Items for Systematic Reviews and Meta-analyses (PRISMA) guidelines. The search was performed on Medline, Embase, Scopus, Web of Science and medRxiv on 30th December 2022. The terms used in the search strings were: ‘Post covid symptom*’, ‘post covid-19 syndrome’, ‘long-COVID’, ‘post-acute covid-19 sequelae’, ‘COVID-19’, ‘SARS-CoV-2’, ‘severe acute respiratory syndrome coronavirus 2’, ‘coronavirus disease 2019’, ‘chronic covid’, ‘post-acute covid syndrome’, ‘post-acute sequelae of SARS-CoV-2 infection’, ‘covid long haulers’, ‘acute COVID-19’, ‘post-COVID conditions’, ‘persistent-covid’, ‘covid complications’, ‘epigenetic’, ‘epigenome’, ‘epigenome-wide association study’, ‘epigenetic modifications’, ‘epigenetic repression’, ‘epigenetic clock’, ‘epigenetic regulation’, ‘miRNA’, ‘microRNA*’, ‘small interfering RNA*’, ‘micro interfering RNA*’, ‘siRNA’, ‘long noncoding RNA*’, ‘noncoding RNA’, ‘lncRNA’, ‘histone modifications’, ‘histone methylation’, ‘histone acetylation’, ‘histone*’, ‘acetylati*’, ‘chromatin remodelling’, ‘chromatin dynamics’, ‘chromatin*’, ‘chromatin reorganization’, ‘nucleosome remodelling’, ‘global methylation’, ‘DNA methylation’, ‘gene methylation’, ‘methylati*’.

The search strings for all five databases and the number of results obtained can be found in the supplementary material (see Table S1 in Supplementary Material). The searches on Medline and Embase were limited to human studies only. No such limit was applied for searches on Web of Science and Scopus. The same keywords were combined and entered in pre-print platform such as medXriv and bioXriv. No publication date and language restrictions were used. We only included articles published in English, Italian and Spanish. Cohort studies, case–controls studies and cross-sectional studies were included. Studies were not selected if they did not include a control group. Only human studies assessing epigenetic biomarkers such as chromatin accessibility, histone modifications, DNA methylation, and miRNA and lncRNA expression in patients with PACS or long-COVID were included. PACS included patients experiencing symptoms persisting more than three weeks from onset; long-COVID included patients experiencing symptoms for at least 12 weeks from onset. Studies assessing epigenetic biomarkers only in acute COVID-19 patients were excluded. In-vitro observations and preclinical studies were also excluded.

### Study selection

After removing the duplicates, the initial screening of studies was done based on titles and abstracts by two reviewers independently (MP and ER). This was done using the Rayyan platform. Any disagreement between the two was resolved by a third reviewer (AP). Afterwards, the full text of the relevant studies was checked for eligibility by the two reviewers independently (MP and ER). In case some of the studies did not report potentially relevant information (e.g. a precise characterization of clinical symptoms reported by patients), we tried to contact the corresponding authors and asked for additional information.

### Data extraction

Studies meeting inclusion criteria were divided between the two reviewers (MP and ER) for independent data extraction, after which both authors checked the extracted data of all the studies and any discrepancies were resolved through discussion. The following data were extracted: first author's name along with the year of publication, study design, assessed epigenetic biomarkers, blood fraction assessed (cells, serum or plasma), type of participants population (PACS or long-COVID), sample characteristics (patients and controls characteristics, sample size, hospitalization status), findings pertaining to the epigenetics of patients and the quality of the study. The following epigenetic outcomes were considered: chromatin accessibility, DNA methylation, miRNA and lncRNA profiling, and histone modifications. For each outcome, studies enrolling patients with either Post-Acute COVID-19 or long-COVID were grouped together.

### Quality assessment

The quality assessment for all the studies was done independently by the two authors (MP and ER) and disagreements were resolved through discussion. Methodological quality was assessed using the NIH study quality assessment tools (https://www.nhlbi.nih.gov/health-topics/study-quality-assessment-tools). We included three additional questions to the ones present in the tool, to include questions regarding confounding variables particularly important for the study of epigenetic biomarkers in the target population and a few other questions regarding the reporting of methods employed. The final criterion for different study designs can be found in the supplementary material (see Table S2). The quality assessment of each study can be found in the supplementary material. An overall rating was given to each study depending on the number of questions with an affirmative answer. Studies with affirmative answers for ⩾ 75% of questions were labelled as good, those with 50–75% were labelled as fair and those with less than 50% were labelled as poor.

## Results

**Figure 1.**
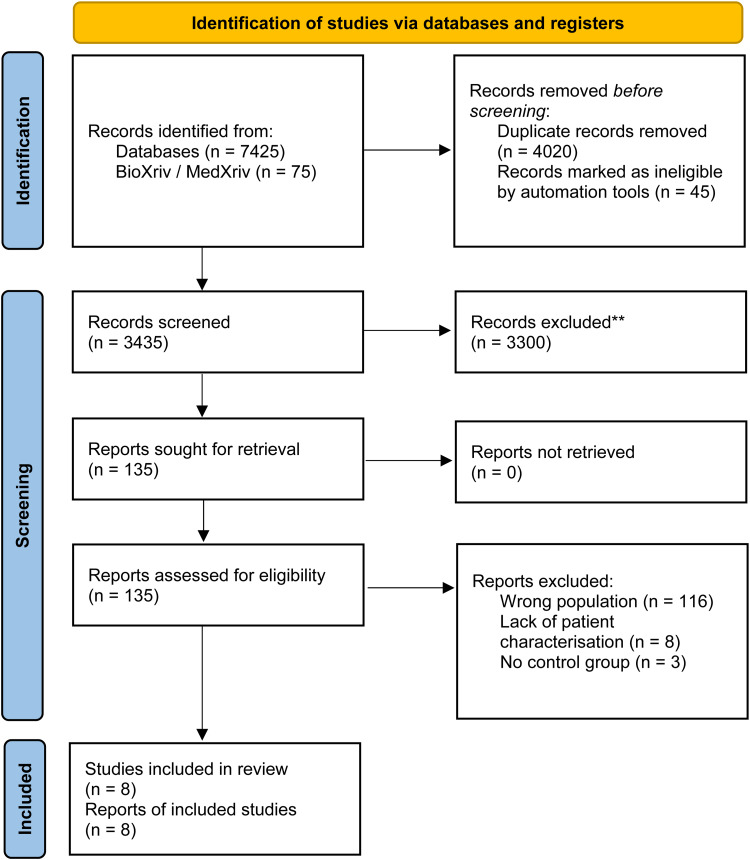
Prisma flowchart of the systematic review.

The initial search on all five databases yielded 7500 potentially relevant studies. Following the removal of duplicates, 3435 studies were screened by title and abstract. After a first screening based on the title and abstract, we excluded 3300 results and screened the full text of 135 studies for inclusion. In total, eight case–control studies matched our inclusion criteria and were included in the final systematic review (Refs 30, 31, 32, 33, 34, 35, 36, 37). The flow diagram of the screening and selection procedure can be found in Figure 1.

### Characteristics of the included studies

**Figure 2.**
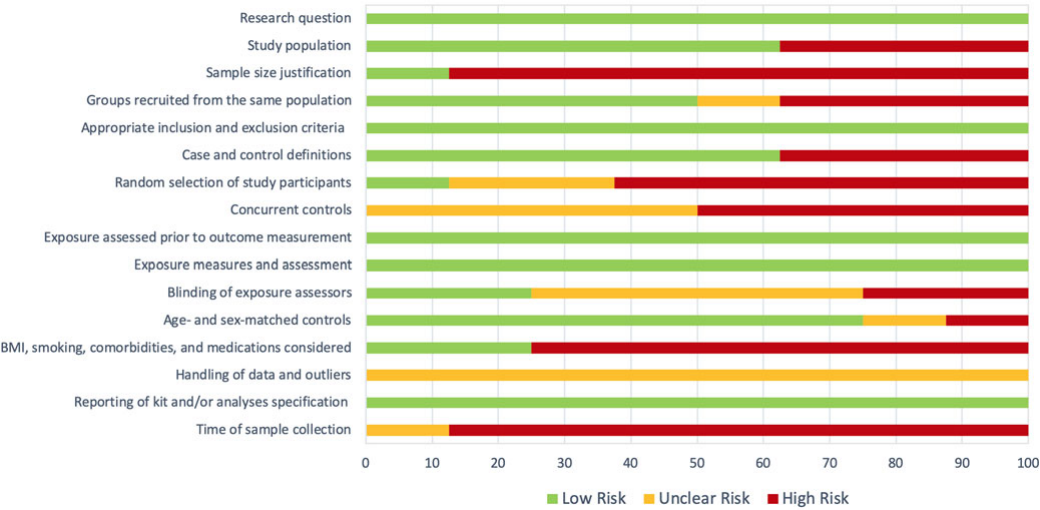
Overall quality of the present systematic review. Risk of bias assessed according to the NIH Risk of bias tools (see supplementary material).

The included articles explored in total 455 patients and 380 controls. Participants were either individuals infected with SARS-CoV-2 who then recovered or healthy controls, with no evidence of infection. The smallest study included 14 participants (Ref. 32), while the largest study included 261 participants (Ref. 30). Five out of eight studies were of fair quality (Refs 31, 33, 34, 35, 37) while three studies were of poor quality (Refs 30, 32, 36). Studied populations were rather heterogeneous. Two studies included participants who were infected at least four weeks before (PACS). The other six studies included participants who were infected three months or more before (long-COVID). In two of these six, persistent symptoms were related to an acute respiratory distress syndrome (ARDS). Two other studies included both subjects with and without evidence of lung complications, in the patient group. No studies explored chromatin accessibility or histone modifications. Six studies explored DNA methylation, (Refs 30, 31, 32, 33, 34, 35) while two studies measured circulating miRNAs, both of them in ARDS-related symptoms (Refs 36, 37). Figure 2 summarizes the overall quality of the present systematic review. For details on the risk of bias assessment for each study, information can be found in Tables S3 (see Supplementary Material).

### Synthesis of the evidence

Given the nature of the data extracted, the heterogeneity of the included samples, the methodological differences, and the different outcome measures, quantitative synthesis using meta-analysis was not possible. Hence, guidelines for synthesis without meta-analysis (SWiM) (Ref. 38) were used to report results in narrative form as an alternative to meta-analysis.

The results are reported in Table 1. Table 1 shows the characteristics of the included studies, and specifies the population included, the findings pertaining to the epigenetics of patients, and the overall study quality, with a comment describing the most relevant criticism of the study.

**Table 1.** Result summary of the systematic review

First author, year	Study design	Population	Sample features	Mechanism / Assessed biomarker	Blood fraction	Epigenetic Method / Assay	Results	Study Quality	Criticism
Mongelli *et al*. (2021) (Ref. 30)	Case–control	PACS	PACS (at least 4 weeks after infection, *N* = 117), age and sex-matched non-infected controls (*N* = 144)	*DNA methylation /* DNA methylation (for epigenetic clock analyses) and telomere length quantification	Whole blood	Pyro-sequencing	Delta age acceleration of 10.45 ± 7.29 years in post-COVID-19 individuals especially younger ones compared to 3.68 ± 8.17 years of COVID-19 free individuals was observed. Average TL was 3.03 ± 2.39 kb in PACS vs. 10.67 ± 11.69 kb in controls.	Poor	Around 50% of patients with PACS showed lung complications (e.g. pneumonia). No symptoms reported.
Huoman *et al*. (2021) (Ref. 31)	Case–control	PACS / long-COVID	PACS or long-COVID (4–24 weeks post-infection, *N* = 14), symptoms free individuals with SARS-CoV-2 specific T cell response (*N* = 6), non-infected healthy controls (*N* = 23).	*DNA methylation /* Epigenome-wide DNA methylation	PBMCs	Illumina MethylationEPIC 850K array	Patients showed a distinct DNA methylome. Differentially methylated CpGs in these patients mapped to 54 unique genes with Wnt and integrin pathways being over-represented. Subsequently, a COVID-19 induced module was identified consisting of 66 genes with Wnt, muscarinic acetylcholine receptor signalling and gonadotropin-releasing hormone receptor pathways being overrepresented.	Fair	Small sample size. Three out of 14 patients included in the PACS/long-COVID group did not report symptoms.
Yin *et al*. (2022) (Ref. 32)	Case–control	PACS/ long-COVID	PACS /long-COVID (2–4 months after infection, n = 9). Age and sex-matched controls (N = 5). Patients were all men, who were hospitalized due to COVID-19.	*DNA methylation /* DNA methylome analysis (whole genome bisulphite sequencing)	PBMCs	Illumina HiSeq 2500 system	Altered expression of DNMT and TET family genes in patients. 18 516 differentially methylated regions (DMRs) in total were found – mainly enriched at gene promoter, intron and other TE regions. Within TE loci, 13 233 DMRs were found: 36.5% in LINE, 38.2% in SINE, 15.7% in LTR and 9.6% in DNA transposons. Other mis-regulated genes were related to immune response, stress response and metabolic processes.	Poor	Small sample size. The more severe cases (*n* = 4) showed signs of pneumonia and/or respiratory tract involvement.
Lee *et al*. (2022) (Ref. 33)	Case–control	PACS / long-COVID	PACS (2–3 months after infection, *n* = 110 with positive RT-PCR), controls (*N* = 74); long-COVID (>3 months after infection, *N* = 41) vs. recovered patients (*N* = 63).	*DNA methylation /* Epigenome-wide association study and detection of epigenetic age acceleration	Whole blood	Illumina Infinium MethylationEPIC BeadChip	The study found three significantly hypomethylated CpGs in PACS compared to controls. Cg22399236 and cg03607951 (located in IFI44L gene), and cg09829636 (located in ANKRD9 gene). No significant differences in DNA methylation were found in long-COVID. No evidence of epigenetic age acceleration was found in either group.	Fair	The control group (*n* = 74) consisted of 41 healthy controls and 32 symptomatic controls with other upper respiratory tract infections. Recovered patients were defined based on one question of the SF-36.
Nikesiö *et al*. (2022) (Ref. 34)	Case–control	Long-COVID	Long-COVID (>12 weeks post-infection, who did not receive hospital care, *N* = 10), recovered, symptoms free patients (*n* = 14), non-infected healthy controls (*N* = 17).	*DNA methylation /* Epigenome-wide DNA methylation	PBMCs	Illumina MethylationEPIC 850K array	Three differentially methylated CpGs (DMCs) were significantly different among all groups and mapped to the SNORD3B, CETP and DLGAP1 genes. Analysis revealed significantly enriched pathways including ‘Angiotensin II-stimulated signalling through G proteins and *β*-arrestin’, ‘Histamine H1 receptor-mediated signalling pathway’, ‘Heterotrimeric G-protein signalling pathway-Gq alpha and Go alpha-mediated pathway’, ‘PI3 kinase pathway’ and ‘Muscarinic acetylcholine receptor 1 and 3 (CHRM1&3) signalling pathway’.	Fair	Small sample size
Balnis *et al*. (2022) (Ref. 35)	Case–control	Long-COVID	Long-COVID (1 year after infection, *n* = 15), pre-pandemic healthy controls (*N* = 39). Patients were hospitalized due to COVID-19.	DNA methylation	Leucocytes	Infinium Human MethylationEPIC 850K BeadChip	64 DMRs were found to be hypermethylated and 7 hypomethylated one year after acute infection. 90% of these were in or near gene promoter regions. These DMRs were included in pathways related to viral responses and inflammation.	Fair	Small sample size.
Ali *et al*. (2022) (Ref. 36)	Case–control	Long-COVID / ARDS	COVID-19 patients 3–6 months after recovery (*N* = 25) and age and sex-matched healthy controls (*N* = 25)	miRNAs	Plasma	RT- qPCR for *miRNA-21*	Patients with long-COVID showed increased levels of miRNA-21 (4.50 ± 1.03 vs 12.60 ± 3.52, *p* < 0.0001), TGF-*β* (0.56 ± 0.27 vs. 1.83 ± 0.98), Col1A2 (0.62 ± 0.19 vs. 1.56 ± 1.00), Col3A1 (0.61 ± 0.27 vs. 1.54 ± 0.89), *α*-SMA (0.46 ± 0.17 vs. 1.20 ± 0.78). miRNA-21 differentiated between groups with >72% sensitivity and >80% specificity.	Poor	No symptoms reported, patients had evidence of persisting lung involvement.
Garcia-Hidalgo *et al*. (2022) (Ref. 37)	Case–control	Long-COVID / ARDS	Long-COVID (>12 weeks after discharge, *n* = 154) meeting the criteria for ARDS; controls who did not develop ARDS (*N* = 33).	miRNAs	Plasma	miRCURY LNA miRNA Custom Panels (41 miRNAs)	Low carbon monoxide diffusion capacity in ARDS patients showed association with miR-17, miR-27a, miR-126, miR-495 and miR-146a. Total severity scores of lung infection were associated with miR-9, miR-24, miR-221 and miR-21. These miRNAs were involved in pathways such as pro-fibrotic states, hypoxia, coagulation, immune response, vascularization, viral infection and cell death.	Fair	Patients had evidence of persisting lung involvement due to ARDS.

### DNA methylation

In total, six studies focussed on DNA methylation changes in people with persistent symptoms after COVID-19. Three of them enrolled patients with a combination of PACS (<12 weeks post-infection) and long-COVID (>12 weeks post infection). Mongelli and colleagues enrolled a group of patients who reported symptoms for at least 4 weeks after infection. They measured DeltaAge – a marker of biological age acceleration based on DNA methylation of specific targeted genes (Ref. 39), telomere length and ACE2 expression in peripheral blood. They found evidence for age acceleration, especially in younger individuals, significant telomere shortening and reduced ACE2 expression (Ref. 30). Huoman *et al*. (2021) (Ref. 31) used a genome-wide approach and identified an overrepresentation of the signalling pathways of Wnt, muscarinic and acetylcholine receptors, and the gonadotropin-releasing hormone pathway (Ref. 31). Yin and colleagues discovered DMRs in transposable elements and genes related to immune and stress responses indicating mild activation of TEs and incomplete recovery of immune-related genes (Ref. 32).

The study by Lee and collegues (Ref. 33) started from a large cohort and performed multiple comparison. One comparison is between patients with PACS (8–12 weeks post infection, with evidence of positive RT-PCR, *n* = 110), and controls (*n* = 73). The control group included both healthy controls and symptomatic patients, who all had a negative RT-PCR and no evidence of COVID-19 persistence. They measured DNA methylation using a genome-wide approach, and epigenetic age acceleration (EAA), according to a published algorithm (Ref. 40). They found three hypomethylated CpGs located in IFI44L and ANKRD9 genes, that are involved in innate immune activation and Class I MHC-mediated antigen processing and presentation, respectively. However, contrary to the findings by Mongelli and colleagues (Ref. 30), they did not find any evidence of age acceleration (Ref. 33). Two main differences should be reported between the two studies. First, Mongelli *et al*. included patients at an earlier stage (from 4 weeks on), while Lee *et al*. included patients with persistent symptoms of at least 8 weeks. Second, some of the patients included in Mongelli *et al*.'s study showed lung involvement. On the contrary, people with respiratory tract infection – not due to COVID-19, were in the control group in Lee's study.

Focussing on long-COVID, Lee *et al*. also compared patients who were symptomatic at least 3 months after infection with participants who were infected but recovered. No between-group differences were found. Of note, patients were assigned to the long-COVID or control group based on one question only of the SF-36 questionnaires, namely *Self-reported health somewhat or much worse than a year-ago.*

The other two studies measuring DNA methylation enrolled patients with long-COVID (>12 weeks after infection). Nikesiö *et al*. (2022) (Ref. 34) found significant differences in DNA methylation in three genes: SNORD3B, CETP and DLGAP1. Such genes are involved in several biological pathways, including ‘Angiotensin II-stimulated signalling through G proteins and *β*-arrestin’, ‘Histamine H1 receptor mediated signalling pathway’, ‘Heterotrimeric G-protein signalling pathway-Gq alpha and Go alpha mediated pathway’, ‘PI3 kinase pathway’ and ‘Muscarinic acetylcholine receptor 1 and 3 (CHRM1&3) signalling pathway’. Balnis and colleagues found 71 DMRs in immune and inflammation-related pathways in long-COVID individuals one year after infection (Ref. 35).

### miRNA expression

Two studies explored miRNA expression and focused on lung and pulmonary abnormalities in long-COVID. One of them found increased levels of miR-21 and gene transcripts such as TGF-*β*, Col1A2, Col3A1 and *α*-SMA involved in profibrotic pathways (Ref. 36). miR-21 had high diagnostic value, as it was able to differentiate between patients and controls >72% sensitivity and >80% specificity. The second one discovered distinct miRNA profiles associated with radiologic features (miR-9, miR-24, miR-221, miR-21) and low CO diffusion (miR-17, miR-27a, miR-126, miR-495, miR-146a) capacity in ARDS patients (Ref. 37).

## Discussion

To our knowledge, the present systematic review is the first attempt to summarize findings on epigenetic changes associated with PACS and long-COVID. The first and most important finding is lack of homogeneity in the patient populations included. The World Health Organisation specifies that long-COVID occurs when symptoms persist for over three months from initial infection and an alternative explanation for those symptoms cannot be found (Ref. 5). However, other groups describe PACS and long-COVID as a multi-organ disease, where evidence of pulmonary, cardiovascular or nervous system alterations are considered part of the disease (Ref. 10). This is important, as some of the included studies included patients with evidence of lung involvement such as ARDS (Refs 36, 37). Though ARDS was a consequence of Sars-CoV-2 infection and persisted for over 12 weeks, it represents a possible explanation for symptoms. Therefore, in such cases the diagnosis of PACS or long-COVID should not be made. In other cases, authors included individuals with and without evidence of pneumonia and/or upper respiratory tract involvement in the same patient group (Refs 30, 32). In these cases, drawing a valid conclusion becomes difficult. A precise reporting of the clinical presentation is crucial – different mechanisms can be at play in different clinical presentations. We urge future research to apply strict inclusion criteria and provide a comprehensive clinical characterization of participants. This would minimize heterogeneity and provide a tailored approach based on mechanism-based subgroups.

Similarly, controls should be precisely defined. In some studies, controls were people who got infected by the virus, but then recovered. In others, controls were healthy participants with no evidence of SARS-CoV-2 infection, or recruited before the COVID-19 pandemic. We argue that the latter control groups are less relevant for studies investigating the development of long-COVID.

The second relevant result is that research focussing on the epigenetics of PACS and long-COVID is scarce and mostly of low quality. Sample sizes are generally quite small, and the clinical description of the included populations is rather superficial. In addition, different studies used different biological matrices and/or methods to explore the same mechanism (e.g. Illumina array-based assays *vs.* whole-genome bisulphite sequencing for DNA methylation). In fact, different studies showed different results, and firm conclusions cannot be drawn on the exact underlying epigenetic mechanisms.

However, the present synthesis of the literature does point towards some epigenetic mechanisms that can be relevant in PACS and long-COVID. With the exception of Mongelli *et al* (Ref. 30), which used pyrosequencing in targeted genes to assess biological age of the participants, the other five studies measuring DNA methylation used array-based technology to study whole-genome DNA methylation. Whole-genome DNA methylation allows for a broad, explorative analyses of the DNA methylome. They require high-level bioinformatic analyses and the results depend at least partially by the algorithm employed for analyses. In addition, the sample size needed to detect small–medium effect sizes is normally very large, and it is not the case for the studies included in the present systematic review. The studies included here were able to only detect changes with large effect size, at best. Precise replication between different studies is unlikely in this scenario. However, the included studies provide some results that are in line with one another. All studies identified significant differential DNA methylation patterns between patients and controls in several genes. The exact patterns differ from study to study but they can be summarized as related to the immune system (IFI44L and ANKRD9 genes, histamine and T-cell activation pathways, etc.), the autonomic nervous system (muscarinic acetylcholine receptor and angiotensin II signalling), and cell metabolism (WNT pathway, DLGAP1 and PI3 kinases, ERK signalling and mitochondrial function). Other changes in DNA methylation are less related to specific mechanisms, but rather represent widespread changes in the epigenome. These are changes in biological age and differential methylation in LINE-1 regions. Finally, patients with PACS might show evidence of biological age acceleration, but this seems be more relevant in PACS associated with pulmonary involvement (Ref. 30), rather than long-COVID (Ref. 33). Taken together, these findings are promising but not conclusive and more research should attempt to replicate them.

One last finding is that individuals with long-COVID due to lung abnormalities show upregulation of miRNAs involved in fibrotic, cell death, vascularization, thrombosis and inflammation-related pathways. ARDS increases pulmonary vascular permeability, inflammation and loss of aerated lung tissue. Dysregulated inflammation along with coagulation and profibrotic pathway activation has been implicated in the pathophysiology of ARDS (Ref. 41). Both studies that investigated long-COVID-associated lung injury assessed miRNA expression (Refs 36, 37). Ali and colleagues focused specifically on circulating levels of miR-21 – a miRNA involved in fibroblast activation and lung fibrosis (Ref. 42) and found it to be significantly increased. Their observation was also corroborated by the study by Garcia-Hidalgo and colleagues in their sample. Though they employed a more explorative approach, including 41 miRNAs in their analyses, they found that higher levels of miR-21 were associated with lung function.

Such findings point towards the importance of patient subgrouping and stratification. When studies focus on homogenous subgroups (in this case patients with long-COVID associated with lung abnormalities), results can be replicated and inform future research. Over 100 symptoms have been reported by patients with long-COVID, making long-COVID a very heterogenous syndrome (Ref. 9). Subgrouping patients based on clinical presentation or putative pathophysiological mechanisms will likely increase the power of statistical analyses and the chances to identify targetable underlying mechanisms. Observations from other populations already showed that epigenetic changes can successfully contribute to stratify patients into more homogeneous subgroups (Ref. 43).

Lastly, we believe it is relevant to note that symptoms of most patients with long-COVID very closely resemble the ones described in another condition – myalgic encephalomyelitis/chronic fatigue syndrome (ME/CFS). ME/CFS is in fact characterized by unexplained, profound fatigue, sleep and cognitive disturbances and PESE (Ref. 44). Many patients suffering from ME/CFS report their symptoms to have started after a viral infection (Ref. 44). The similarity is striking. Research on long-COVID can learn a lot from the knowledge obtained from studies on ME/CFS over the past decades (Ref. 45). In turn, current research on long-COVID will benefit patients with ME/CFS (Ref. 46). Given the expected huge prevalence of long-COVID (and thus the possibility to recruit patients for research purposes), results from studies on patients with long-COVID should be widely transferred to the ME/CFS field (Ref. 46).

The findings of this review should be considered in light of its limitations. As previously mentioned, sample sizes were often small and study quality was generally low. Heterogeneity in methods and sample characteristics exist among the included studies. The timing of the follow-up and assessment is also crucial, especially for studying DNA methylation, which is time-dependent and decreases with age. Individual variabilities in disease presentation, the stage of recovery, and variability in the follow-up measurements make it hard to understand pathophysiological mechanisms and their association to disease progression.

In addition, there are various important covariates that might affect both the disease trajectory and epigenetic mechanisms which are not always controlled for in the studies. Age, sex, medication used, comorbidities such as diabetes, hypertension, smoking, obesity, etc. may alter epigenetics, especially DNA methylation and should be controlled for (Refs 47, 48, 49). Moreover, the treatment patients received during their acute infection might play a role in deciding the trajectory of these patients and must be considered during statistical analysis (Ref. 50). Finally, all included studies were case–control studies, and cross-sectional in nature. While such designs can provide relevant information, longitudinal studies are better at characterizing the dynamics of epigenetics and should be preferred.

## Conclusion

Epigenetic changes might help the understanding of PACS and long-COVID, elucidate their underlying mechanisms, aid subgrouping and point towards tailored treatments. Preliminary evidence is promising but scarce. We urge future research to employ longitudinal, repeated-measures designs, aim for the best possible methodological quality (following published guidelines on conducting and reporting clinical research, e.g (Ref. 51) or https://www.equator-network.org/), and provide a comprehensive, precise clinical characterization of the included population in order to provide valid and reliable findings. A better understanding of PACS and long-COVID is urgently needed, given the high prevalence of these conditions and the benefits that such research can transfer to other high-burden diseases such as ME/CFS.

## Supporting information

Shekhar Patil et al. supplementary material Shekhar Patil et al. supplementary material

## Data Availability

Not applicable.

## Supplementary material

For supplementary material accompanying this paper visit http://doi.org/10.1017/erm.2024.32. click here to view supplementary material
