# Supplementary material for: Epigenetic changes in patients with post-acute COVID-19 symptoms (PACS) and long-COVID: A systematic review
Source: Expert Rev Mol Med. 2024 Oct 22;26:e29. doi: 10.1017/erm.2024.32 (PMC11505605; doi:10.1017/erm.2024.32)
Supplement: Shekhar Patil et al. supplementary material [file S1462399424000322sup001.docx]

**SUPPLEMENTARY MATERIAL**

**Table S1: Search strings and number of results for every database**

| **DATABASE** | **SEARCH STRING** | **RESULTS** |
| --- | --- | --- |
| **PubMed** | (("post"[All Fields] AND ("sars cov 2"[MeSH Terms] OR "sars cov 2"[All Fields] OR "covid"[All Fields] OR "covid 19"[MeSH Terms] OR "covid 19"[All Fields]) AND "symptom*"[All Fields]) OR ("post"[All Fields] AND ("covid 19"[All Fields] OR "covid 19"[MeSH Terms] OR "covid 19 vaccines"[All Fields] OR "covid 19 vaccines"[MeSH Terms] OR "covid 19 serotherapy"[All Fields] OR "covid 19 serotherapy"[Supplementary Concept] OR "covid 19 nucleic acid testing"[All Fields] OR "covid 19 nucleic acid testing"[MeSH Terms] OR "covid 19 serological testing"[All Fields] OR "covid 19 serological testing"[MeSH Terms] OR "covid 19 testing"[All Fields] OR "covid 19 testing"[MeSH Terms] OR "sars cov 2"[All Fields] OR "sars cov 2"[MeSH Terms] OR "severe acute respiratory syndrome coronavirus 2"[All Fields] OR "ncov"[All Fields] OR "2019 ncov"[All Fields] OR (("coronavirus"[MeSH Terms] OR "coronavirus"[All Fields] OR "cov"[All Fields]) AND 2019/11/01:3000/12/31[Date - Publication])) AND ("syndrom"[All Fields] OR "syndromal"[All Fields] OR "syndromally"[All Fields] OR "syndrome"[MeSH Terms] OR "syndrome"[All Fields] OR "syndromes"[All Fields] OR "syndrome s"[All Fields] OR "syndromic"[All Fields] OR "syndroms"[All Fields])) OR ("post acute covid 19 syndrome"[Supplementary Concept] OR "post acute covid 19 syndrome"[All Fields] OR "long covid"[All Fields]) OR ("post-acute"[All Fields] AND ("covid 19"[All Fields] OR "covid 19"[MeSH Terms] OR "covid 19 vaccines"[All Fields] OR "covid 19 vaccines"[MeSH Terms] OR "covid 19 serotherapy"[All Fields] OR "covid 19 serotherapy"[Supplementary Concept] OR "covid 19 nucleic acid testing"[All Fields] OR "covid 19 nucleic acid testing"[MeSH Terms] OR "covid 19 serological testing"[All Fields] OR "covid 19 serological testing"[MeSH Terms] OR "covid 19 testing"[All Fields] OR "covid 19 testing"[MeSH Terms] OR "sars cov 2"[All Fields] OR "sars cov 2"[MeSH Terms] OR "severe acute respiratory syndrome coronavirus 2"[All Fields] OR "ncov"[All Fields] OR "2019 ncov"[All Fields] OR (("coronavirus"[MeSH Terms] OR "coronavirus"[All Fields] OR "cov"[All Fields]) AND 2019/11/01:3000/12/31[Date - Publication])) AND ("complications"[MeSH Subheading] OR "complications"[All Fields] OR "sequelae"[All Fields] OR "sequela"[All Fields] OR "sequelaes"[All Fields] OR "sequelas"[All Fields])) OR ("covid 19"[All Fields] OR "covid 19"[MeSH Terms] OR "covid 19 vaccines"[All Fields] OR "covid 19 vaccines"[MeSH Terms] OR "covid 19 serotherapy"[All Fields] OR "covid 19 serotherapy"[Supplementary Concept] OR "covid 19 nucleic acid testing"[All Fields] OR "covid 19 nucleic acid testing"[MeSH Terms] OR "covid 19 serological testing"[All Fields] OR "covid 19 serological testing"[MeSH Terms] OR "covid 19 testing"[All Fields] OR "covid 19 testing"[MeSH Terms] OR "sars cov 2"[All Fields] OR "sars cov 2"[MeSH Terms] OR "severe acute respiratory syndrome coronavirus 2"[All Fields] OR "ncov"[All Fields] OR "2019 ncov"[All Fields] OR (("coronavirus"[MeSH Terms] OR "coronavirus"[All Fields] OR "cov"[All Fields]) AND 2019/11/01:3000/12/31[Date - Publication])) OR ("sars cov 2"[MeSH Terms] OR "sars cov 2"[All Fields] OR "sars cov 2"[All Fields]) OR ("sars cov 2"[MeSH Terms] OR "sars cov 2"[All Fields] OR "severe acute respiratory syndrome coronavirus 2"[All Fields]) OR ("covid 19"[MeSH Terms] OR "covid 19"[All Fields] OR "coronavirus disease 2019"[All Fields]) OR (("chronic"[All Fields] OR "chronical"[All Fields] OR "chronically"[All Fields] OR "chronicities"[All Fields] OR "chronicity"[All Fields] OR "chronicization"[All Fields] OR "chronics"[All Fields]) AND ("sars cov 2"[MeSH Terms] OR "sars cov 2"[All Fields] OR "covid"[All Fields] OR "covid 19"[MeSH Terms] OR "covid 19"[All Fields])) OR ("post acute covid 19 syndrome"[Supplementary Concept] OR "post acute covid 19 syndrome"[All Fields] OR "post acute covid syndrome"[All Fields]) OR ("post acute covid 19 syndrome"[Supplementary Concept] OR "post acute covid 19 syndrome"[All Fields] OR "post acute sequelae of sars cov 2 infection"[All Fields]) OR (("sars cov 2"[MeSH Terms] OR "sars cov 2"[All Fields] OR "covid"[All Fields] OR "covid 19"[MeSH Terms] OR "covid 19"[All Fields]) AND "long"[All Fields] AND "haulers"[All Fields]) OR ("post acute covid 19 syndrome"[Supplementary Concept] OR "post acute covid 19 syndrome"[All Fields] OR "long haul covid"[All Fields]) OR (("acute"[All Fields] OR "acutely"[All Fields] OR "acutes"[All Fields]) AND ("covid 19"[All Fields] OR "covid 19"[MeSH Terms] OR "covid 19 vaccines"[All Fields] OR "covid 19 vaccines"[MeSH Terms] OR "covid 19 serotherapy"[All Fields] OR "covid 19 serotherapy"[Supplementary Concept] OR "covid 19 nucleic acid testing"[All Fields] OR "covid 19 nucleic acid testing"[MeSH Terms] OR "covid 19 serological testing"[All Fields] OR "covid 19 serological testing"[MeSH Terms] OR "covid 19 testing"[All Fields] OR "covid 19 testing"[MeSH Terms] OR "sars cov 2"[All Fields] OR "sars cov 2"[MeSH Terms] OR "severe acute respiratory syndrome coronavirus 2"[All Fields] OR "ncov"[All Fields] OR "2019 ncov"[All Fields] OR (("coronavirus"[MeSH Terms] OR "coronavirus"[All Fields] OR "cov"[All Fields]) AND 2019/11/01:3000/12/31[Date - Publication]))) OR ("post"[All Fields] AND ("sars cov 2"[MeSH Terms] OR "sars cov 2"[All Fields] OR "covid"[All Fields] OR "covid 19"[MeSH Terms] OR "covid 19"[All Fields]) AND ("condition s"[All Fields] OR "conditions"[All Fields] OR "disease"[MeSH Terms] OR "disease"[All Fields] OR "condition"[All Fields])) OR (("persist"[All Fields] OR "persistance"[All Fields] OR "persistant"[All Fields] OR "persisted"[All Fields] OR "persistence"[All Fields] OR "persistences"[All Fields] OR "persistencies"[All Fields] OR "persistency"[All Fields] OR "persistent"[All Fields] OR "persistently"[All Fields] OR "persistents"[All Fields] OR "persister"[All Fields] OR "persisters"[All Fields] OR "persisting"[All Fields] OR "persists"[All Fields]) AND ("sars cov 2"[MeSH Terms] OR "sars cov 2"[All Fields] OR "covid"[All Fields] OR "covid 19"[MeSH Terms] OR "covid 19"[All Fields])) OR (("sars cov 2"[MeSH Terms] OR "sars cov 2"[All Fields] OR "covid"[All Fields] OR "covid 19"[MeSH Terms] OR "covid 19"[All Fields]) AND ("complicances"[All Fields] OR "complicate"[All Fields] OR "complicated"[All Fields] OR "complicates"[All Fields] OR "complicating"[All Fields] OR "complication"[All Fields] OR "complication s"[All Fields] OR "complications"[MeSH Subheading] OR "complications"[All Fields]))) **AND** ("epigenetical"[All Fields] OR "epigenetically"[All Fields] OR "epigenomics"[MeSH Terms] OR "epigenomics"[All Fields] OR "epigenetic"[All Fields] OR "epigenetics"[All Fields] OR ("epigenome"[MeSH Terms] OR "epigenome"[All Fields] OR "epigenomes"[All Fields] OR "epigenomically"[All Fields] OR "epigenomics"[MeSH Terms] OR "epigenomics"[All Fields] OR "epigenomic"[All Fields]) OR (("epigenome"[MeSH Terms] OR "epigenome"[All Fields] OR "epigenomes"[All Fields] OR "epigenomically"[All Fields] OR "epigenomics"[MeSH Terms] OR "epigenomics"[All Fields] OR "epigenomic"[All Fields]) AND "wide"[All Fields] AND ("associate"[All Fields] OR "associated"[All Fields] OR "associates"[All Fields] OR "associating"[All Fields] OR "association"[MeSH Terms] OR "association"[All Fields] OR "associations"[All Fields]) AND ("studies"[All Fields] OR "study"[All Fields] OR "study s"[All Fields] OR "studying"[All Fields] OR "studys"[All Fields])) OR (("epigenetical"[All Fields] OR "epigenetically"[All Fields] OR "epigenomics"[MeSH Terms] OR "epigenomics"[All Fields] OR "epigenetic"[All Fields] OR "epigenetics"[All Fields]) AND ("modification"[All Fields] OR "modifications"[All Fields])) OR ("epigenetic repression"[MeSH Terms] OR ("epigenetic"[All Fields] AND "repression"[All Fields]) OR "epigenetic repression"[All Fields]) OR (("epigenetical"[All Fields] OR "epigenetically"[All Fields] OR "epigenomics"[MeSH Terms] OR "epigenomics"[All Fields] OR "epigenetic"[All Fields] OR "epigenetics"[All Fields]) AND ("clock"[All Fields] OR "clock s"[All Fields] OR "clocked"[All Fields] OR "clocking"[All Fields] OR "clocks"[All Fields])) OR (("epigenetical"[All Fields] OR "epigenetically"[All Fields] OR "epigenomics"[MeSH Terms] OR "epigenomics"[All Fields] OR "epigenetic"[All Fields] OR "epigenetics"[All Fields]) AND ("legislation and jurisprudence"[MeSH Subheading] OR ("legislation"[All Fields] AND "jurisprudence"[All Fields]) OR "legislation and jurisprudence"[All Fields] OR "regulations"[All Fields] OR "social control, formal"[MeSH Terms] OR ("social"[All Fields] AND "control"[All Fields] AND "formal"[All Fields]) OR "formal social control"[All Fields] OR "regulate"[All Fields] OR "regulates"[All Fields] OR "regulating"[All Fields] OR "regulation s"[All Fields] OR "regulative"[All Fields] OR "regulator"[All Fields] OR "regulator s"[All Fields] OR "regulators"[All Fields] OR "regulated"[All Fields] OR "regulation"[All Fields])) OR ("micrornas"[MeSH Terms] OR "micrornas"[All Fields] OR "mirna"[All Fields] OR "mirnas"[All Fields] OR "mirna s"[All Fields]) OR ("microrna s"[All Fields] OR "micrornas"[MeSH Terms] OR "micrornas"[All Fields] OR "microrna"[All Fields]) OR ("rna, small interfering"[MeSH Terms] OR ("rna"[All Fields] AND "small"[All Fields] AND "interfering"[All Fields]) OR "small interfering rna"[All Fields] OR ("small"[All Fields] AND "interfering"[All Fields] AND "rna"[All Fields])) OR (("micro"[All Fields] OR "micros"[All Fields]) AND ("interfered"[All Fields] OR "interfering"[All Fields]) AND ("rna"[MeSH Terms] OR "rna"[All Fields])) OR ("rna, small interfering"[MeSH Terms] OR ("rna"[All Fields] AND "small"[All Fields] AND "interfering"[All Fields]) OR "small interfering rna"[All Fields] OR "sirna"[All Fields] OR "sirna s"[All Fields] OR "sirnas"[All Fields]) OR ("rna, long noncoding"[MeSH Terms] OR ("rna"[All Fields] AND "long"[All Fields] AND "noncoding"[All Fields]) OR "long noncoding rna"[All Fields] OR ("long"[All Fields] AND "noncoding"[All Fields] AND "rna"[All Fields])) OR ("rna, untranslated"[MeSH Terms] OR ("rna"[All Fields] AND "untranslated"[All Fields]) OR "untranslated rna"[All Fields] OR ("noncoding"[All Fields] AND "rna"[All Fields]) OR "noncoding rna"[All Fields]) OR ("lncrnas"[All Fields] OR "rna, long noncoding"[MeSH Terms] OR ("rna"[All Fields] AND "long"[All Fields] AND "noncoding"[All Fields]) OR "long noncoding rna"[All Fields] OR "lncrna"[All Fields]) OR ("histone code"[MeSH Terms] OR ("histone"[All Fields] AND "code"[All Fields]) OR "histone code"[All Fields] OR ("histone"[All Fields] AND "modifications"[All Fields]) OR "histone modifications"[All Fields]) OR (("histon"[All Fields] OR "histones"[MeSH Terms] OR "histones"[All Fields] OR "histone"[All Fields] OR "histonic"[All Fields] OR "histons"[All Fields]) AND ("methyl"[All Fields] OR "methylate"[All Fields] OR "methylated"[All Fields] OR "methylates"[All Fields] OR "methylating"[All Fields] OR "methylation"[MeSH Terms] OR "methylation"[All Fields] OR "methylations"[All Fields] OR "methylational"[All Fields] OR "methylator"[All Fields] OR "methylators"[All Fields] OR "methyls"[All Fields])) OR (("histon"[All Fields] OR "histones"[MeSH Terms] OR "histones"[All Fields] OR "histone"[All Fields] OR "histonic"[All Fields] OR "histons"[All Fields]) AND ("acetyl"[All Fields] OR "acetylate"[All Fields] OR "acetylated"[All Fields] OR "acetylates"[All Fields] OR "acetylating"[All Fields] OR "acetylation"[MeSH Terms] OR "acetylation"[All Fields] OR "acetylations"[All Fields] OR "acetyls"[All Fields])) OR "acetylati*"[All Fields] OR ("chromatin assembly and disassembly"[MeSH Terms] OR ("chromatin"[All Fields] AND "assembly"[All Fields] AND "disassembly"[All Fields]) OR "chromatin assembly and disassembly"[All Fields] OR ("chromatin"[All Fields] AND "remodelling"[All Fields]) OR "chromatin remodelling"[All Fields]) OR (("chromatin"[MeSH Terms] OR "chromatin"[All Fields] OR "chromatins"[All Fields] OR "chromatin s"[All Fields] OR "chromatine"[All Fields] OR "chromatinization"[All Fields] OR "chromatinized"[All Fields]) AND ("dynamer"[All Fields] OR "dynamers"[All Fields] OR "dynamic"[All Fields] OR "dynamical"[All Fields] OR "dynamically"[All Fields] OR "dynamicity"[All Fields] OR "dynamics"[All Fields] OR "dynamism"[All Fields] OR "dynamisms"[All Fields])) OR "chromatin*"[All Fields] OR (("chromatin"[MeSH Terms] OR "chromatin"[All Fields] OR "chromatins"[All Fields] OR "chromatin s"[All Fields] OR "chromatine"[All Fields] OR "chromatinization"[All Fields] OR "chromatinized"[All Fields]) AND ("organisation"[All Fields] OR "organization and administration"[MeSH Subheading] OR ("organization"[All Fields] AND "administration"[All Fields]) OR "organization and administration"[All Fields] OR "organization"[All Fields] OR "organizations"[MeSH Terms] OR "organizations"[All Fields] OR "organisation s"[All Fields] OR "organisational"[All Fields] OR "organisations"[All Fields] OR "organise"[All Fields] OR "organised"[All Fields] OR "organiser"[All Fields] OR "organisers"[All Fields] OR "organises"[All Fields] OR "organising"[All Fields] OR "organization s"[All Fields] OR "organizational"[All Fields] OR "organizations s"[All Fields] OR "organize"[All Fields] OR "organized"[All Fields] OR "organizer"[All Fields] OR "organizer s"[All Fields] OR "organizers"[All Fields] OR "organizes"[All Fields] OR "organizing"[All Fields])) OR (("nucleosomal"[All Fields] OR "nucleosomally"[All Fields] OR "nucleosome s"[All Fields] OR "nucleosomes"[MeSH Terms] OR "nucleosomes"[All Fields] OR "nucleosome"[All Fields] OR "nucleosomic"[All Fields]) AND ("remodel"[All Fields] OR "remodelation"[All Fields] OR "remodeled"[All Fields] OR "remodeler"[All Fields] OR "remodelers"[All Fields] OR "remodeling"[All Fields] OR "remodelings"[All Fields] OR "remodelled"[All Fields] OR "remodeller"[All Fields] OR "remodellers"[All Fields] OR "remodelling"[All Fields] OR "remodellings"[All Fields] OR "remodels"[All Fields])) OR (("global"[All Fields] OR "globalism"[All Fields] OR "globalize"[All Fields] OR "globalized"[All Fields] OR "globalizes"[All Fields] OR "globalizing"[All Fields] OR "globally"[All Fields] OR "globals"[All Fields] OR "internationality"[MeSH Terms] OR "internationality"[All Fields] OR "globalization"[All Fields]) AND ("methyl"[All Fields] OR "methylate"[All Fields] OR "methylated"[All Fields] OR "methylates"[All Fields] OR "methylating"[All Fields] OR "methylation"[MeSH Terms] OR "methylation"[All Fields] OR "methylations"[All Fields] OR "methylational"[All Fields] OR "methylator"[All Fields] OR "methylators"[All Fields] OR "methyls"[All Fields])) OR ("dna methylation"[MeSH Terms] OR ("dna"[All Fields] AND "methylation"[All Fields]) OR "dna methylation"[All Fields]) OR (("genes"[MeSH Terms] OR "genes"[All Fields] OR "gene"[All Fields]) AND ("methyl"[All Fields] OR "methylate"[All Fields] OR "methylated"[All Fields] OR "methylates"[All Fields] OR "methylating"[All Fields] OR "methylation"[MeSH Terms] OR "methylation"[All Fields] OR "methylations"[All Fields] OR "methylational"[All Fields] OR "methylator"[All Fields] OR "methylators"[All Fields] OR "methyls"[All Fields])) OR "methylati*"[All Fields])  **AND** ("human s"[All Fields] OR "humans"[MeSH Terms] OR "humans"[All Fields] OR "human"[All Fields]) | **1,691** |
| **WEB OF SCIENCE** | ALL=((“Post covid symptom*” OR “Post covid-19 syndrome” OR “Long covid” OR “Post-acute covid-19 sequelae” OR “COVID-19” OR “SARS-CoV-2” OR “Severe acute respiratory syndrome coronavirus 2” OR “Coronavirus disease 2019” OR “Chronic covid” OR “Post-acute covid syndrome” OR “Post-acute sequelae of SARS-CoV-2 infection” OR “Covid long haulers” OR “Long-haul covid” OR “Acute covid-19” OR “Post-covid conditions” OR “Persistent-covid” OR “Covid complications”) **AND** (“Epigenetic modifications” OR “Epigenome wide association study” OR “Epigenome” OR “Epigenetic” OR “Epigenetic repression” OR “Epigenetic clock” OR “Epigenetic regulation” OR “miRNA” OR “microRNA*” OR “small interfering RNA*” OR “micro interfering RNA” OR “siRNA” OR “long noncoding RNA*” OR “noncoding RNA” OR “lncRNA” OR “histone modifications” OR “histone methylation” OR “histone acetylation” OR “histone*” OR “acetylati*” OR “chromatin remodelling” OR “chromatin dynamics” OR “chromatin*” OR “chromatin organization” OR “nucleosome remodelling” OR “global methylation” OR “DNA methylation” OR “Gene methylation” OR “Methylati*”)) | **1,772** |
| **EMBASE** | ('post covid symptom*' OR 'post covid-19 syndrome'/exp OR 'post covid-19 syndrome' OR 'long covid'/exp OR 'long covid' OR 'post-acute covid-19 sequelae' OR 'covid-19'/exp OR 'covid-19' OR 'sars-cov-2'/exp OR 'sars-cov-2' OR 'severe acute respiratory syndrome coronavirus 2'/exp OR 'severe acute respiratory syndrome coronavirus 2' OR 'coronavirus disease 2019'/exp OR 'coronavirus disease 2019' OR 'chronic covid' OR 'post-acute covid syndrome'/exp OR 'post-acute covid syndrome' OR 'post-acute sequelae of sars-cov-2 infection' OR 'covid long haulers' OR 'long-haul covid'/exp OR 'long-haul covid' OR 'acute covid-19' OR 'post-covid conditions' OR 'persistent-covid' OR 'covid complications') **AND** ('epigenetic modifications' OR 'epigenome wide association study'/exp OR 'epigenome wide association study' OR 'epigenome'/exp OR 'epigenome' OR 'epigenetic'/exp OR 'epigenetic' OR 'epigenetic repression'/exp OR 'epigenetic repression' OR 'epigenetic clock'/exp OR 'epigenetic clock' OR 'epigenetic regulation'/exp OR 'epigenetic regulation' OR 'mirna'/exp OR 'mirna' OR 'microrna*' OR 'small interfering rna*' OR 'micro interfering rna' OR 'sirna'/exp OR 'sirna' OR 'long noncoding rna*' OR 'noncoding rna'/exp OR 'noncoding rna' OR 'lncrna'/exp OR 'lncrna' OR 'histone modifications' OR 'histone methylation'/exp OR 'histone methylation' OR 'histone acetylation'/exp OR 'histone acetylation' OR 'histone*' OR 'acetylati*' OR 'chromatin remodelling'/exp OR 'chromatin remodelling' OR 'chromatin dynamics'/exp OR 'chromatin dynamics' OR 'chromatin*' OR 'chromatin organization'/exp OR 'chromatin organization' OR 'nucleosome remodelling' OR 'global methylation' OR 'dna methylation'/exp OR 'dna methylation' OR 'gene methylation'/exp OR 'gene methylation' OR 'methylati*') **AND** [humans]/lim | **2,598** |
| **SCOPUS** | TITLE-ABS-KEY ( "Post covid symptom*"  OR  "Post covid-19 syndrome"  OR  "Long covid"  OR  "Post-acute covid-19 sequelae"  OR  "COVID-19"  OR  "SARS-CoV-2"  OR  "Severe acute respiratory syndrome coronavirus 2"  OR  "Coronavirus disease 2019"  OR  "Chronic covid"  OR  "Post-acute covid syndrome"  OR  "Post-acute sequelae of SARS-CoV-2 infection"  OR  "Covid long haulers"  OR  "Long-haul covid"  OR  "Acute covid-19"  OR  "Post-covid conditions"  OR  "Persistent-covid"  OR  "Covid complications" )  **AND**  TITLE-ABS-KEY ( "Epigenetic modifications"  OR  "Epigenome wide association study"  OR  "Epigenome"  OR  "Epigenetic"  OR  "Epigenetic repression"  OR  "Epigenetic clock"  OR  "Epigenetic regulation"  OR  "miRNA"  OR  "microRNA*"  OR  "small interfering RNA*"  OR  "micro interfering RNA"  OR  "siRNA"  OR  "long noncoding RNA*"  OR  "noncoding RNA"  OR  "lncRNA"  OR  "histone modifications"  OR  "histone methylation"  OR  "histone acetylation"  OR  "histone*"  OR  "acetylati*"  OR  "chromatin remodelling"  OR  "chromatin dynamics"  OR  "chromatin*"  OR  "chromatin organization"  OR  "nucleosome remodelling"  OR  "global methylation"  OR  "DNA methylation"  OR  "Gene methylation"  OR  "Methylati*" ) | **2,398** |

**Table S2: Modified NIH Quality assessment tool for Case-control Studies**

|  | **The methodological quality of Case-Control Studies** | **Yes** | **No** | **Other**  **(CD, NR, NA)** | **Comment** |
| --- | --- | --- | --- | --- | --- |
| 1 | Was the research question or objective in this paper clearly stated and appropriate? |  |  |  |  |
| 2 | Was the study population clearly specified and defined?   - Acceptable definition of cases and controls - Demographics, location, time period mentioned? - Same characteristics as the target population? |  |  |  |  |
| 3 | Did the authors include a sample size justification?   - Info about the statistical power of the study and sample size calculation |  |  |  |  |
| 4 | Were controls selected or recruited from the same or similar population that gave rise to the cases (including the same timeframe and community/hospital)? |  |  |  |  |
| 5 | Were the definitions, inclusion and exclusion criteria, algorithms, or processes used to identify or select cases and controls valid, reliable, and implemented consistently across all study participants?   - Use of the same underlying criteria for all groups (same age, sex, etc) - Were the inclusion and exclusion criteria developed prior to recruitment? |  |  |  |  |
| 6 | Were the cases clearly defined and differentiated from controls?   - Clear definition of cases (confirmed by +ve RT-PCR / antibodies / radiological signs [Chest X-ray and CT] / COVID-19(post-COVID) symptoms/hospitalization records/status [mild/moderate/severe]) - Clear definition of controls |  |  |  |  |
| 7 | If less than 100 percent of eligible cases and/or controls were selected for the study, were the cases and/or controls randomly selected from those eligible?   - Consecutive sampling for prospective case-control studies? |  |  |  |  |
| 8 | Was there use of concurrent controls? |  |  |  |  |
| 9 | Were the investigators able to confirm that the exposure/risk occurred prior to the development of the condition or event that defined a participant as a case? |  |  |  |  |
| 10 | Were the measures of exposure/risk clearly defined, valid, reliable, (not self-reported), and implemented consistently across all study participants?   - Tools (RT-PCR / antibodies / radiological signs / COVID-19(post-COVID) symptoms / hospital records) |  |  |  |  |
| 11 | Were the assessors of exposure/risk blinded to the case or control status of participants? (Potential bias if not blinded) |  |  |  |  |
| 12 | Were the patients and HCs age and sex-matched or statistically controlled? If matching was used, did the investigators account for matching during the study analysis? |  |  |  |  |
| 13 | Were other potential confounding variables measured and adjusted, e.g. BMI, smoking, comorbidities, and medications? |  |  |  |  |
| 14 | Reporting how data under detection limit and outliers were handled? |  |  |  |  |
| 15 | Reporting of either the manufacturer of the test or its parameters (detection limit and coefficient of variation)? |  |  |  |  |
| 16 | Was the time of sample collection specified and/or controlled for (e.g. morning vs. evening; fasting vs. not fasting)? |  |  |  |  |

**CD = cannot determine, NA = not applicable, NR = not reported**

| **Table S3. RISK OF BIAS ASSESSMENT**  Assessment based on the NIH assessment tools, all information and guidelines can be found here:  <https://www.nhlbi.nih.gov/health-topics/study-quality-assessment-tools> | | | | | | | | | | | | | | | | | |
| --- | --- | --- | --- | --- | --- | --- | --- | --- | --- | --- | --- | --- | --- | --- | --- | --- | --- |
|  | Research question | Study population | Sample size justification | Groups recruited from the same population | Appropriate inclusion and exclusion criteria | Case and control definitions | Random selection of study participants | Concurrent controls | Exposure assessed prior to outcome measurement | Exposure measures and assessment | Blinding of exposure assessors | Age- and sex-matched controls | BMI, smoking, comorbidities, etc. considered | Handling of data and outliers | Reporting of kit and/or analyses specification | Time of sample collection | **Overall validity** |
| **Mongelli et al., 2021** | 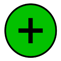 | 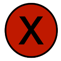 | 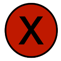 | 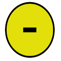 | 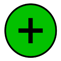 | 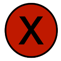 | 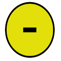 | 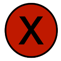 | 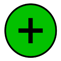 | 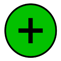 | 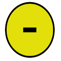 | 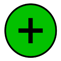 | 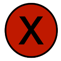 | 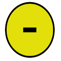 | 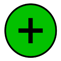 | 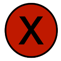 | **Poor** |
| **Huoman et al., 2021** | 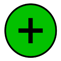 | 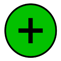 | 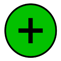 | 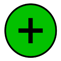 | 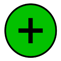 | 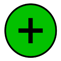 | 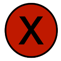 | 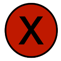 | 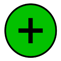 | 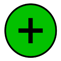 | 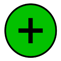 | 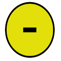 | 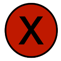 | 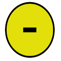 | 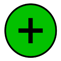 | 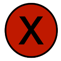 | **Fair** |
| **Nikesiö et al., 2022** | 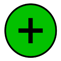 | 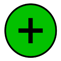 | 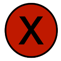 | 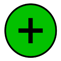 | 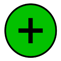 | 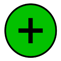 | 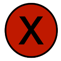 | 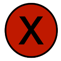 | 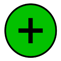 | 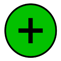 | 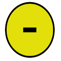 | 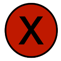 | 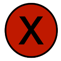 | 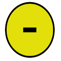 | 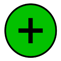 | 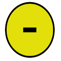 | **Fair** |
| **Yin et al., 2022** | 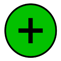 | 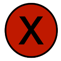 | 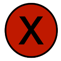 | 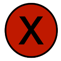 | 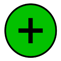 | 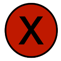 | 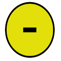 | 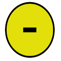 | 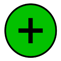 | 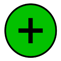 | 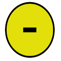 | 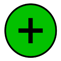 | 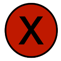 | 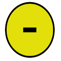 | 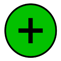 | 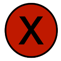 | **Poor** |
| **Lee et al., 2022** | 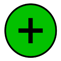 | 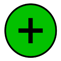 | 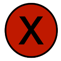 | 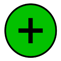 | 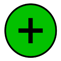 | 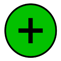 | 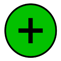 | 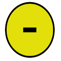 | 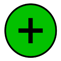 | 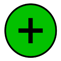 | 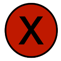 | 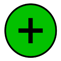 | 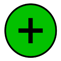 | 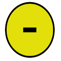 | 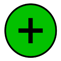 | 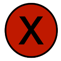 | **Fair** |
| **Balnis et al., 2022** | 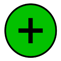 | 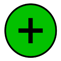 | 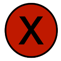 | 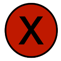 | 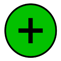 | 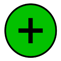 | 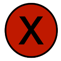 | 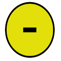 | 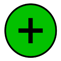 | 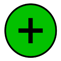 | 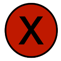 | 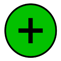 | 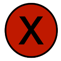 | 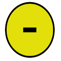 | 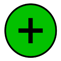 | 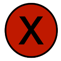 | **Fair** |
| **Ali et al., 2022** | 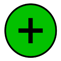 | 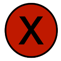 | 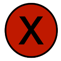 | 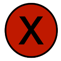 |  |  |  |  |  |  |  |  |  |  |  |  | **Poor** |
| **Garcia-Hidalgo et al., 2022** |  |  |  |  |  |  |  |  |  |  |  |  |  |  |  |  | **Fair** |
|  |  |  |  |  |  |  |  |  |  |  |  |  |  |  |  |  |  |
|  |  |  |  |  |  |  |  |  |  |  |  |  |  |  |  |  |  |

|  | Comments |
| --- | --- |
| Mongelli et al., 2021 | No exact location and time period given. No sample size justification given.  No mention of whether assessors were blinded. Age and sex- considered in the analyses, no mention of other covariates. |
| Huoman et al., 2021 | Consecutive sampling. No mention of whether assessors were blinded.  Age and sex- reported, but unclear if included in the analyses, no mention of other covariates. |
| Nikesiö et al., 2022 | No sample size justification given. Consecutive sampling. No mention of whether assessors were blinded. Age and sex-matching mentioned, but results show lack of matching. Unclear whether covariates were considered in statistical models. |
| Yin et al., 2022 | No time period given. No sample size justification given. No mention of whether assessors were blinded.  Age and sex- considered in the analyses, no mention of other covariates. |
| Lee et al., 2022 | No sample size justification. |
| Balnis et al., 2022 | No sample size justification. Pre-pandemic controls included as a control group. |
| Ali et al., 2022 | No demographics, location, and time period given. No sample size justification given. No time period given. Consecutive sampling. No mention of whether assessors were blinded. Age and sex- considered in the analyses, no mention of other covariates. |
| Garcia-Hidalgo et al., 2022 | No sample size justification given. Consecutive sampling. Age and sex- considered in the analyses.  Smoking, pulmonary disease, and use of corticosteroids as covariates in statistical analysis |
